# Dyads or quads? Impact of group size and learning context on collaborative learning

**DOI:** 10.3389/fpsyg.2023.1168208

**Published:** 2023-05-05

**Authors:** Mindi Wang, Ling Jiang, Heng Luo

**Affiliations:** Faculty of Artificial Intelligence in Education, Central China Normal University, Wuhan, China

**Keywords:** collaborative learning, group size, learning context, learning outcome, learning engagement, collaborative experience

## Abstract

Collaborative learning has been widely used in both offline and online contexts to support deep learning, and its effectiveness may be adjusted by the size of the collaborative groups. To examine the effect of learning context and group size on collaborative learning, this study conducted two experiments with 62 third-year undergraduate students enrolled in the course named *Application of Modern Educational Technology* to compare learning outcomes, learning engagement, and collaborative experience between quad (four-person) and dyad groups in both face-to-face and online learning contexts. The results indicated that learning outcomes and collaborative experience were not significantly affected by group size and learning context, but for peer interaction, the dyad group showed more communication and interaction during the learning process. In general, the dyad group showed higher and more stable scores in all aspects, as well as being able to adapt to changes in learning contexts. Based on the research results, three practical implications were proposed to promote the implementation of collaborative learning in teaching.

## 1. Introduction

Collaborative learning has been widely used in various disciplinary fields in both face-to-face and online learning contexts. The literature has affirmed the advantages of collaborative learning in traditional classrooms: it promotes improvement in students’ higher-order thinking ability, such as problem-solving, critical thinking, and creative thinking (Segundo Marcos et al., 2020; Liang et al., 2021), and also provides students with improved learning experience and superior learning outcomes (Laal and Ghodsi, 2012). Many researchers have also reported the benefits of collaborative learning in online learning environments. For example, Dewiyanti et al. (2007) found that students had a more positive collaborative experience and greater satisfaction in an asynchronous online environment. Similarly, Strauß and Rummel (2020) proved that integrating collaborative learning into online courses is beneficial to students’ learning performance and social presence. Synthesizing the results from many empirical studies, several meta-analyses have confirmed the positive impact of collaborative learning across various educational contexts (Ibrahim and Harun, 2015; Chen et al., 2018; Kaldırım and Tavşanlı, 2018; Talan, 2021).

While the literature supports the overall effectiveness of collaborative learning, its effectiveness is largely dependent upon specific instructional strategies such as task design, role scripts, prework activities, and group size (Pfister and Oehl, 2009; Luo et al., 2023). Group size merits particular attention due to its ease of implementation and great influence on social interaction (Kim, 2013; Wang et al., 2020). Generally, small groups consist of 14 or fewer members, medium groups consist of 15–34 members, and large groups have a minimum size of 35 members (Benton et al., 2015; Parks-Stamm et al., 2017). The literature has identified benefits and limitations for each group size during collaboration: large or medium groups can produce more diverse perspectives but are susceptible to social loafing (Qiu and McDougall, 2015; Amichai-Hamburger et al., 2016), while small groups tend to promote dyadic communication and individual contributions but can be heavily influenced by group dynamics and relationships (Kim, 2013; Yang et al., 2022). Overall, the empirical evidence supports the superiority of small groups over large or medium groups in promoting collaborative learning behaviors, experiences, and achievements (Shaw, 2013; Luo et al., 2023).

However, the optimal group size for collaborative learning within the small range has not yet been identified in the literature. While some researchers have shown that students in dyad groups outperformed those in groups of three or four (Lohman and Finkelstein, 2000; Kim et al., 2020), other studies found that quad (four-person) groups functioned better than dyads in terms of learning performance and social discourse (Peltokorpi and Niemi, 2019; Corrégé and Michinov, 2021). Additionally, the optimal group size might vary with different disciplinary domains. For instance, groups of two or three members were found to be superior in technology courses such as robotics and artificial intelligence (Bianco, 2014; Zhan et al., 2022) while groups of five demonstrated enhanced social interactions in English courses (Abuseileek, 2012). Furthermore, the findings regarding group size are known to be moderated by the type of classroom settings where collaborative learning occurs (Corrégé and Michinov, 2021); however, few studies have systematically compared the effect of group size between the offline and online learning contexts, and little is known about the optimal group size for each contextual setting.

To address this research need, we conducted two experimental studies in an undergraduate course with 62 third-year students randomly assigned to quad and dyad groups. One experiment took place in a face-to-face offline learning context, while the other experiment occurred in an online learning context. The purpose of the study was to determine the impact of group size (four person vs. dyad) on collaborative learning in both offline and online contexts and to explore the moderating effect of learning context. More specifically, we sought to answer the following research questions:

1. Do collaborative learning outcomes differ between quad and dyad groups in offline and online learning contexts?
2. Does collaborative learning engagement differ between quad and dyad groups in offline and online learning contexts?
3. Do collaborative experiences differ between quad and dyad groups in offline and online learning contexts?
4. What are the moderating effects of learning context on students’ learning outcomes, learning engagement, and collaborative experiences in both quad and dyad groups?

## 2. Literature review

### 2.1. Collaborative learning

Learning occurs in the social process of knowledge construction, not as a single effort (Vygotsky, 1978). Collaborative learning, as a learning approach, allows students to organize activities together in the form of groups, forming a small learning community in which everyone has shared responsibility and power to construct knowledge and meaning (Moreira, et al., 2016). Collaborative learning also provides teachers with activities and strategies to facilitate student-centered learning, and it is widely accepted and practiced in both face-to-face and online contexts. According to Dillenbourg (1999), collaborative learning describes “a situation in which particular forms of interaction among people are expected to occur, which would trigger learning mechanisms” (p. 5). Wang and Liao (2017) emphasized the constructive nature of collaborative learning and argued that it enables knowledge construction through mutual participation and motivation of group members. Gu et al. (2015) further identified two key conditions of effective collaborative learning: positive interdependence and individual accountability through which all members contribute to the meaning making and knowledge sharing process. Synthesizing the existing definitions in the literature, in this study, we denote collaborative learning as two or more students completing learning tasks and solve learning problems together through mutual participation, division of labor, and coordinated efforts.

In recent years, with the development of internet technology, collaborative learning has also been applied to the online learning environment. The advantages, challenges, and solutions in the application of collaborative learning in the new environment have formed a new research field: computer-supported collaborative learning (CSCL). The main differences between CSCL and traditional collaborative learning are location (online virtual environment) and time (both synchronous and asynchronous). However, even if the form of collaborative learning changes, its characteristics and purpose are to promote learning outcomes (Shen and Wu, 2011; Wu, 2018), to create better learning engagement (Gopinathan et al., 2022) and to provide a collaborative experience (Burgess et al., 2014; Liu et al., 2017). These three components are interrelated. Higher learning engagement and a positive collaborative experience promote improvements in learning outcomes, which in turn affect learning engagement and the collaborative experience. At present, many scholars have also used social network analysis to explore peer interaction in the process of collaboration, but we believe that interaction in the process of collaboration is not the purpose of collaboration, but rather a means to enable learners to obtain better results and experience.

### 2.2. Effect of group size in the offline context

In our studies, offline environment refers to face-to-face teaching in the real world. In this environment, peer discussion and social interaction play a crucial role in the process of collaboration. When carrying out collaborative learning, we usually ask, “How many people are in the optimally sized group?” No consensus has, however, been reached on the answer to this question. Research has shown that students prefer smaller groups, including peer teaching. For example, Kooloos et al. (2011) divided a 15-person group into three groups of five people; they achieved higher learning gains, greater goal achievement, and a more active sense of participation. Slavin (1987) found that the results of paired learning were better than those for groups of four or more people. Other studies have tried to distinguish the differences between dyads and three-person groups (Jensen and Wiley, 2006; Zhan et al., 2022). Jensen and Wiley (2006) showed that in the task of solving arithmetic problems, three-person groups showed better reasoning ability and more effective password problem solving ability than the dyads. In artificial intelligence courses, Zhan et al. (2022) determined that the dyads were better than the three-person groups in learning motivation and quality of collaborative problem solving over the 6-week course, and also imposed more cognitive load on the individual students. There have, however, also been studies that suggest a group of four people is the optimal group size (Alexopoulou and Driver, 1996; Sung et al., 2017).

### 2.3. Effect of group size in the online context

With the rapid advancement of internet and mobile technologies, online learning is becoming increasingly popular in higher education and has emerged as a major context for CSCL (Hmelo-Silver and Jeong, 2021; Gopinathan et al., 2022). The literature has also explored the effect of group size on online CSCL. A recent meta-analysis revealed a significant influence for group size on online problem-based learning (Saqr et al., 2019): larger groups tended to lead to weaker the team cohesion, worse individual performance, and less social interaction. Through asymmetric collaborative simulation, Rannastu et al. (2019) found that dyads reported better collaboration than quads in grade six. Similarly, several researchers have found that small groups of three members led to slightly better participation and performance in online discussion (Shaw, 2013; Luo et al., 2023).

Similar to the offline environment, there have also been studies that show that a group of four people is ideal (Sugai et al., 2018) for using social networks for collaborative argumentation. Compared with offline environment, however, online collaborative learning faces some challenges. Due to the lack of facial expressions, intonation, and gestures in the online environment, the dialogue between members is more likely to be misunderstood, and they need more time and energy to maintain communication effectiveness. The online environment is also more likely to foster social loafing, even as it provides convenience in time and place through asynchronous communication.

To sum up, we have reason to believe that group size in collaborative learning tends to be more collaborative in small groups in either offline or online environments, and this may be related to the relationship between individual responsibility and motivation. In group decision-making, individual input is often lower than in individual decision-making. Dyads have an equal relationship in the process of collaboration, have more opportunities to participate, and team members can concentrate on thinking without being influenced by other members (Dugosh et al., 2000). The quad group is still small in scale, but it can increase the diversity of views and mutual feedback while also minimizing the possibility of “free riding” (Shimazoe and Aldrich, 2010). Therefore, this study used the group sizes of two and four people per group and compared the changes in offline and online learning contexts to determine the impact of different group sizes on collaborative learning in different learning contexts.

## 3. Methods

### 3.1. Participants

The present study was conducted in the first 4 weeks of a 7-week blended course named *Application of Modern Educational Technology* offered by a research university in central China. A total of 62 third-year students agreed to participate; participants were from the undergraduate programs of chemistry (teacher training) and mathematics (teacher training). All of the participants were randomly assigned into eight quad groups (*n* = 32) and 15 dyad groups (*n* = 30). All of the groups had participated in two sessions of collaborative learning in both offline and online learning contexts, each lasting for 2 weeks. The gender ratio was roughly 7.9:1 (55 female students and seven male students), and the mean age was 20.32 (min = 19, max = 23). The research protocols and instruments were reviewed and approved by the Institutional Review Board of Central China Normal University (CCNU-IRB-202103019, approved on 2021/03/16). Informed consent was obtained from all of the participants before implementing the study.

### 3.2. Procedure

**Figure 1:**
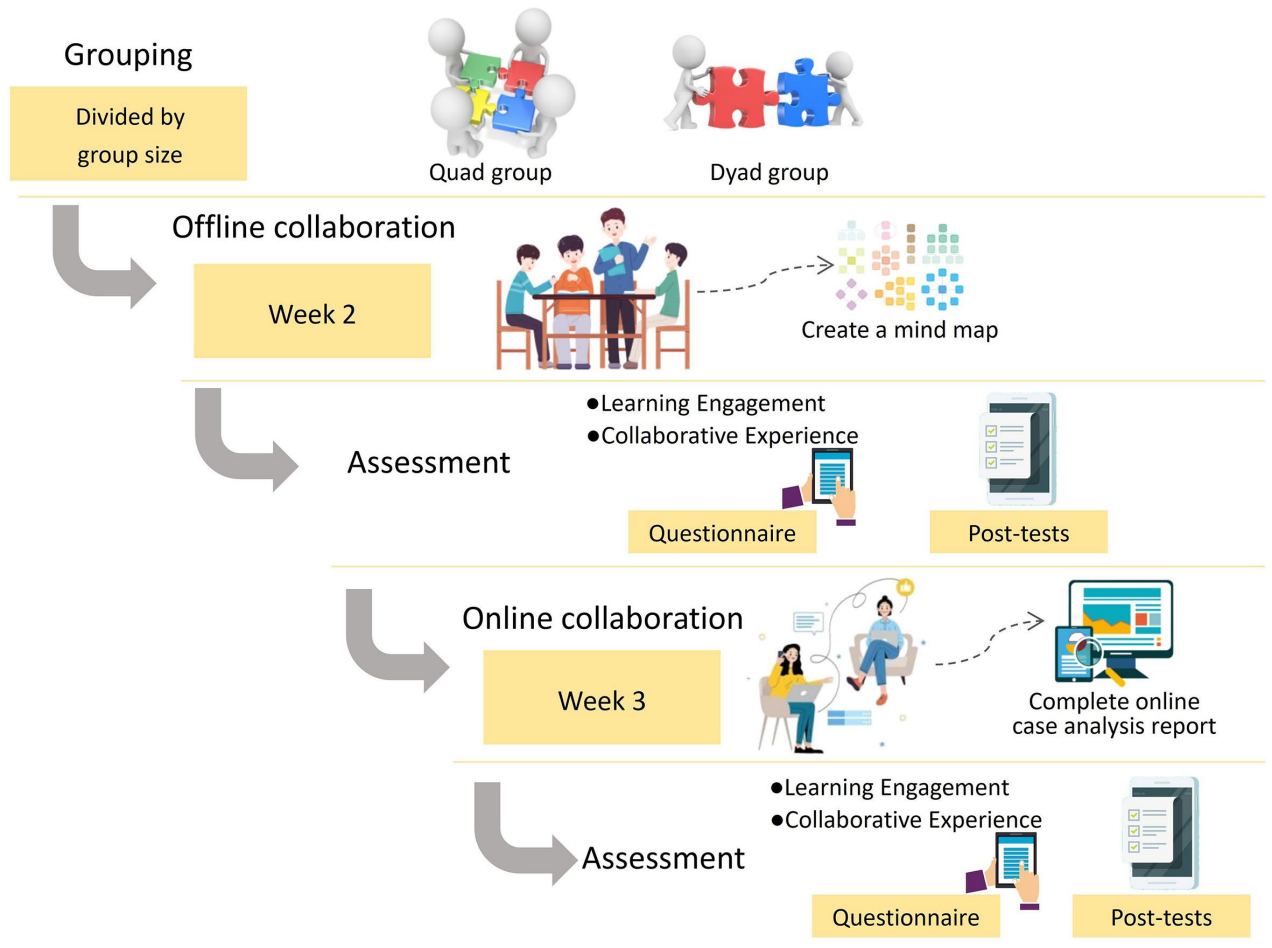
Experimental process.

The entire experimental process is shown in Figure 1. In the first week of the course, the teacher gave an overview of the course and explained the basic information such as the course requirements and assessment methods. A total of 62 students were randomly divided into eight quad groups (*n* = 32) and 15 dyad groups (*n* = 30). In the second week, all of the participants worked in groups offline to complete a collaborative learning assignment that involved the creation of a mind map while attending a face-to-face class. The offline collaboration session lasted about 45 min. In the third week, the participants switched to the online learning mode and worked together with their group members to study and discuss a learning design case in an online discussion forum. The online collaboration session was asynchronous and lasted for 6 days. Afterward, all of the participants were required to submit a case analysis report. Both the offline and online tasks tested the participants’ comprehension of leaning and teaching theories and their higher-order thinking skills such as application, analysis, and creation. At the end of each collaborative learning session, the participants were required to complete a learning experience questionnaire and submit the task assignment (i.e., mind map or case analysis report) individually.

**Figure 2:**
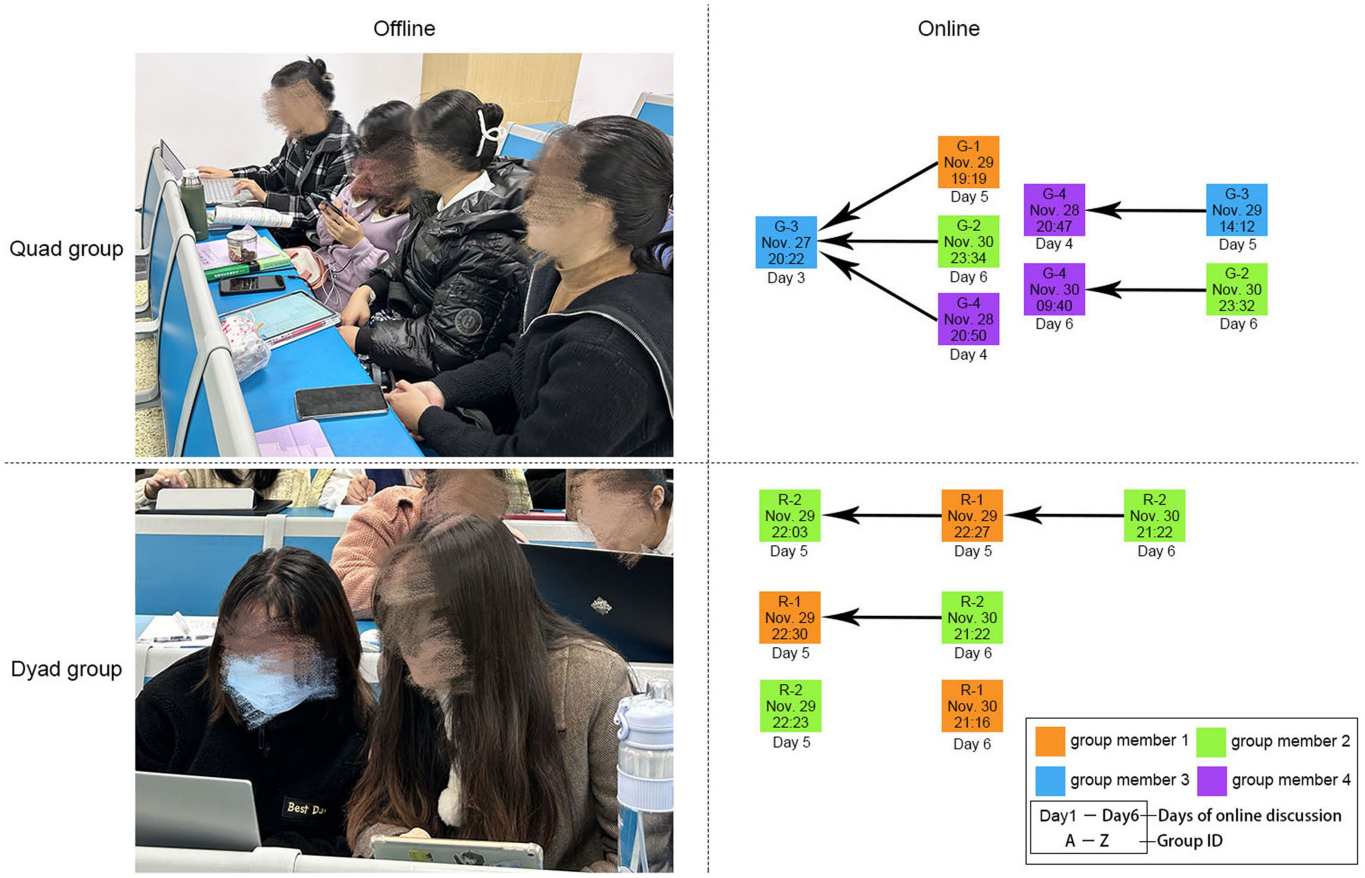
Offline and online collaborative learning.

Figure 2 shows how the participants collaborated with their group members in both offline and online contexts. In the offline collaboration, the group members sit adjacently in one row for convenient communication, and used various electronic devices such as tablets, mobile phones, and laptops to jointly complete the mind map assignment. In the online collaboration, the social interactions among the group members took mainly the form of discussion threads. As seen in Figure 2, the majority of the messages were posted in the latter phase of the discussion period with few threads extended over three interactions; there were also many instances of “orphan” postings that received no replies.

It is important to note that, although this experiment has two independent variables: group size (quad group vs. dyad group) and learning context (offline vs. online), we decided to investigate their effect on collaborative learning separately rather than simultaneously through a two-by-two factorial design. There are two reasons behind this decision: First, synchronous face-to-face communication and asynchronous online communication are inherently different featured by varying activity length and interaction intensity, and the cofounding influence of such differences cannot be eliminated from the two-by-two design. Second, the focus of the present study is on comparing the effectiveness of dyad and quad group, with learning contexts being examined as a possible moderating factor. Consequently, we believed that conducting two separate experiments in each learning context can better answer our research questions and provide more flexibility in research design such as choosing different collaboration task or collaboration length.

### 3.3. Data collection and instruments

The data were collected using four instruments: a learning experience questionnaire, a post-test on learning and teaching theory, grading criteria for the collaborative learning assignment, and a semi-structured interview protocol.

#### 3.3.1. Learning experience questionnaire

The learning experience questionnaire comprised three parts. The first part included five items about personal information: name, student ID, gender, age, and group size. The second part was a subscale measuring learning engagement in terms of cognitive, social, and behavioral engagement; it included 17 items that were informed by the works of Fredricks et al. (2004), Gunuc and Kuzu (2015), and Ma and Zhou (2019). The third part was a subscale measuring collaborative experience, which included 14 items that were informed by the works of Barron (2000) and Miao et al. (2017). All of the subscale items were scored on a five-point Likert scale ranging from 1, “strongly disagree,” to 5 “strongly agree.” The complete questionnaire items are shown in Supplementary Appendix A. During the research process, the subscales for learning engagement and collaborative experience were tested twice. The Cronbach’s α values for learning engagement were 0.777 and 0.927, and for collaborative experience they were 0.867 and 0.950, which indicated good internal reliability.

#### 3.3.2. Post-tests for collaborative learning

The research group developed two post-tests to measure students’ collaborative learning outcomes. The first one was given at the end of the offline collaboration session focusing on learning theories (e.g., behaviorist, cognitive, and socio-cognitive theories), and the second one was given at the end of the online collaboration session focusing on teaching theories (e.g., nine events of instruction, first principles of instruction, and community of inquiry framework). Both tests consisted of 20 multiple-choice questions with a maximum total score of 100 points (five points for each question). Despite the different testing content, the two post-tests were considered equivalent in test format, structure, and difficulty.

#### 3.3.3. Grading criteria for collaborative learning assignment

During the entire experiment, students from different groups completed two collaborative learning assignments in different learning context—the production of mind maps and the writing of online case analysis reports. The former focused on learning theory and the latter on teaching theory. Students’ learning outcomes could be measured through the analysis of the two assignments. The two collaborative learning assignments were independently scored by four researchers according to three aspects. The scoring criteria for the mind map were correctness (correctly expressing multiple concepts and their relationships related to the subject, without obvious intellectual errors); comprehensiveness (reflecting the relevant content of the subject as fully as possible); and rendering effect (concision or selecting and refining keywords, clarity of structure, and the rational use of symbols and diagrams). The scoring criteria for the case analysis reports were correctness (correct expression of the problems in the case without obvious intellectual errors); comprehensiveness (reflecting the teaching problems in the case as fully as possible); and quality of the description (proper number of words, in-depth analysis of the problems and solutions, and the level of detail of the description). The interrater reliability Spearman’s rho was greater than 0.9.

#### 3.3.4. Interview protocol

To understand how students’ collaborative learning experience varied by group size and learning context, we conducted semi-structured interviews with 10 selected participants immediately after the second experiment to gather their in-depth opinions about the contextualized collaboration in terms of perceived benefits, challenges, peer- and self-evaluation, and improvement suggestions, as well as their preference for group size and learning context. A total of 110 min of interview data were recorded and transcribed for future analysis. Please refer to Supplementary Appendix B for details about the interview protocol.

### 3.4. Data analysis

Two rounds of one-way ANOVA were conducted to investigate the differences in participants’ collaborative learning outcomes, leaning engagement, and collaborative experience between quad and dyad groups in both offline and online learning contexts. The Kolmogorov–Smirnov test and Levene’s test were conducted to ensure that the statistical assumptions of normality and homogeneity were met for ANOVA. Quantitative data were analyzed using IBM SPSS (version 23). Interview data were analyzed qualitatively to extract themed findings that would enable triangulation and meaningful interpretation of the statistical results.

## 4. Results

### 4.1. Learning outcomes

**Figure 3:**
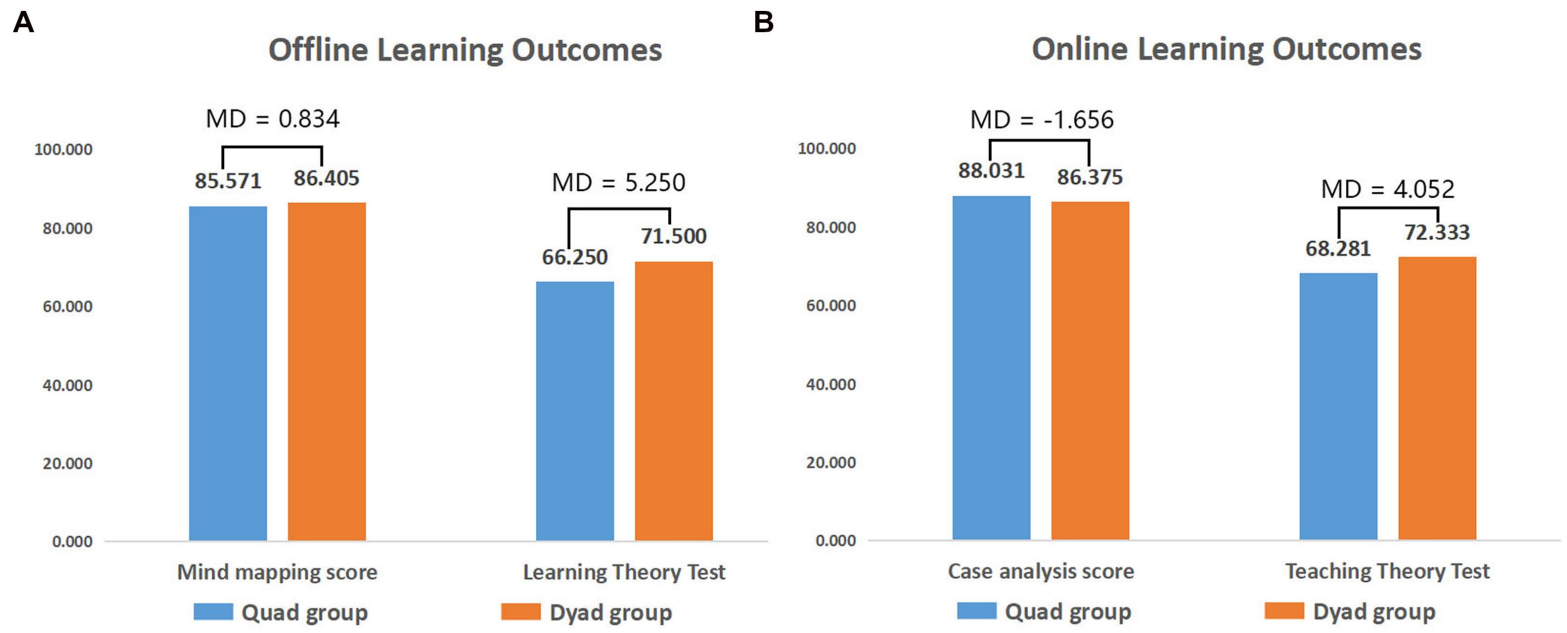
Learning outcomes in different learning contexts. **(A)** Offline learning outcomes; **(B)** Online learning outcomes. MD = mean difference (dyad group score − quad group score).

Students’ learning outcomes were reflected by the average scores for the collaborative learning assignments (full score 100) and post-tests (full score 100). As shown in Figure 3, the offline collaborative learning assignment was the production of a mind map, and the post-tests covered learning theory; the online collaborative learning assignments and post-tests were the online case analysis reports and teaching theory tests, respectively. The data in the figure illustrate that there seems to be no difference in the learning outcomes between the quad and dyad groups, in both online and offline learning. Although there was a gap of 5.25 and 4.052 in the post-tests—that is, the scores for the dyad group were slightly higher—the *t*-test results showed that there was no significant difference. From the figure, we can also see that the quad group showed improvement in the online learning condition in terms of both the collaborative learning assignments and post-tests, but there was little difference in the scores of the dyad groups. It is possible that there were, however, more changes in the learning process; as one dyad collaborative student noted: “In fact, the final results may not be much different, but the process may be different.”

### 4.2. Learning engagement

**Figure 4:**
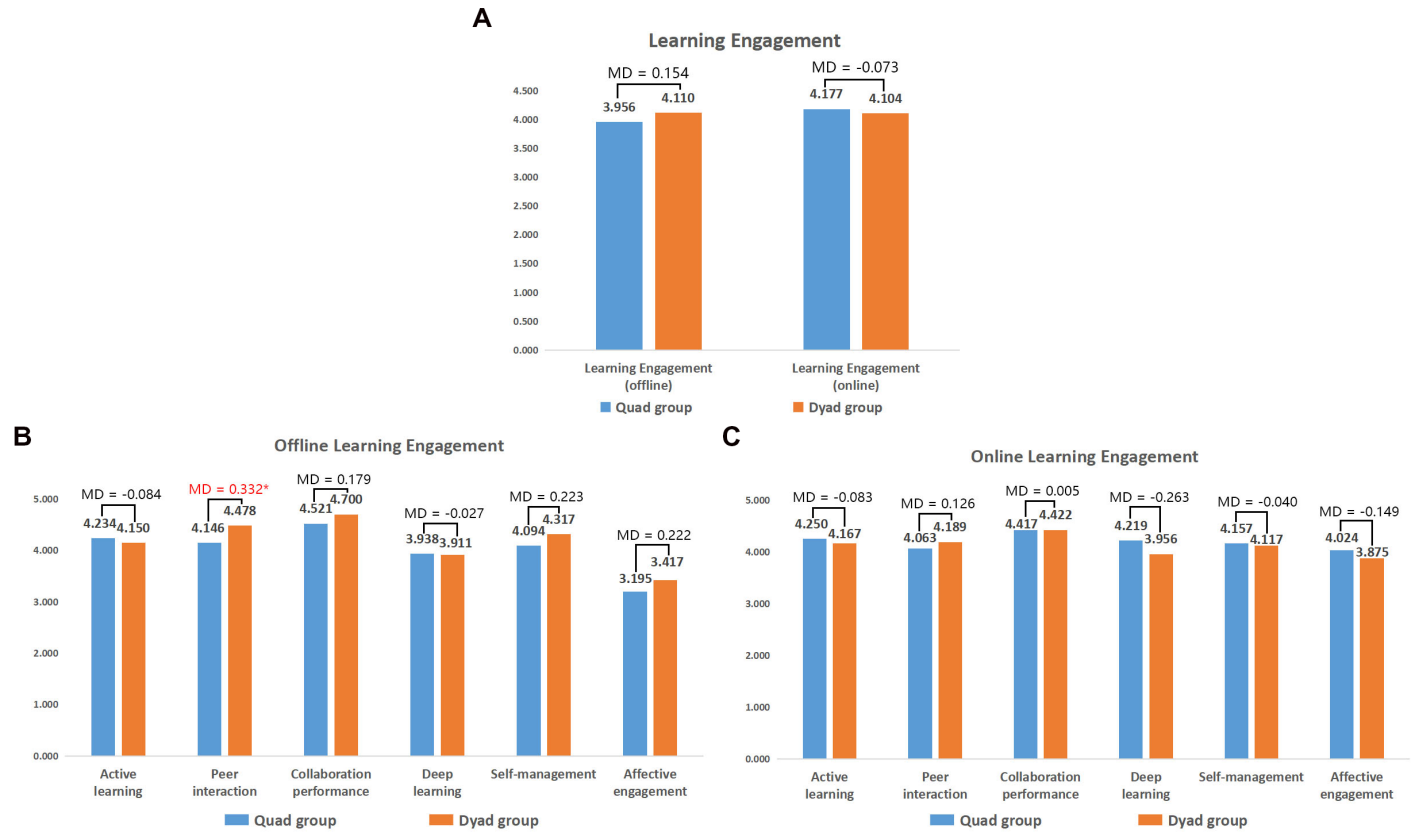
Learning engagement in different learning contexts. **(A)** Total score of offline and online learning engagement; **(B)** Offline learning engagement; **(C)** Online learning engagement. MD, mean difference (dyad group score − quad group score); ^*^p < 0.05; ^**^p < 0.01.

We compared the learning engagement of students with different collaboration modes in the learning process and plotted the average scores in Figure 4. The difference was statistically significant and marked with an asterisk. The information in the figure indicates that there is little difference in learning engagement between students in the quad and dyad groups. However, after further analysis, as shown in Figure 4B, we found that although the dyad groups had fewer group members, they reported more communication and mutual assistance with peers during the offline face-to-face collaborative learning process than the quad groups (MD = 0.332, *F* = 3.078, *p* = 0.014 < 0.05). This indicates that increasing the number of collaborators does not necessarily lead to more communication and exchange within the group. This also explains, to some extent, the reason why the dyads performed better in comprehensiveness (MD = 4.033) in the production of the mind map for the offline collaborative learning assignments. In online learning, although the results showed no significant difference, the dyads still had more communication and mutual assistance than the quad groups (MD = 0.126).

### 4.3. Collaborative experience

**Figure 5:**
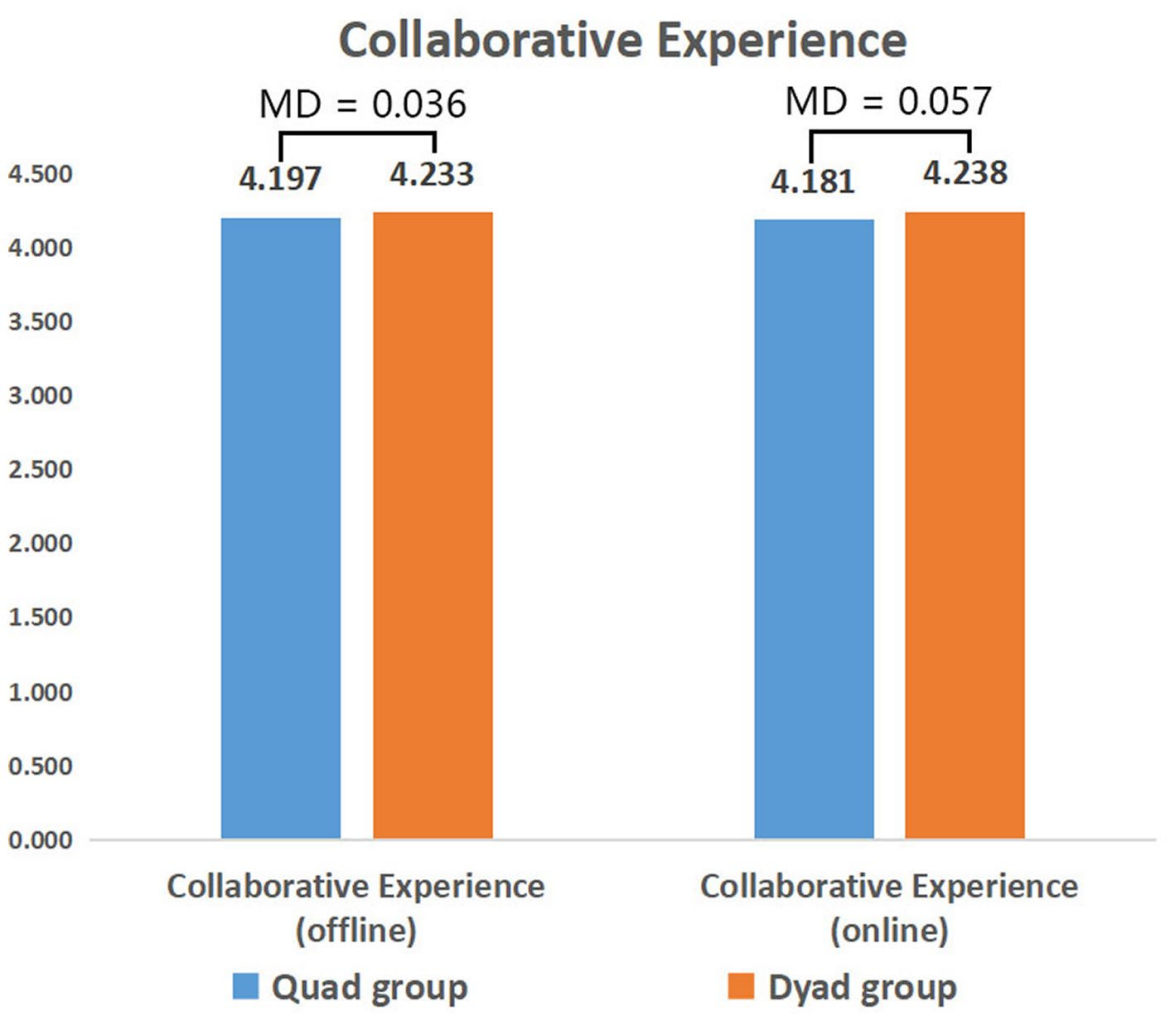
Collaborative experience in different learning context. MD = mean difference (dyad group score − quad group score); ^*^p < 0.05; ^**^p < 0.01.

Figure 5 shows the average scores for the collaborative experience of students in different-sized groups in the learning process. In general, participants had a positive attitude toward collaborative learning regardless of the size of their collaboration groups, as there was no significant difference in their perceived collaborative experience. Most participants believed that collaborative learning could promote understanding of course content (Item 33, M = 4.270), stimulate their own interest in learning (Item 34, M = 4.035), and expressed their willingness to participate in collaborative learning if they had the opportunity (Item 36, M = 4.040). Interestingly, the item with the lowest score in the two questionnaires was students’ interest in collaborative pedagogy (Item 20, M = 3.280), which indicates that although participants had a positive attitude toward collaborative learning, it is still not very attractive to them.

### 4.4. Difference between offline and online collaboration

**Figure 6:**
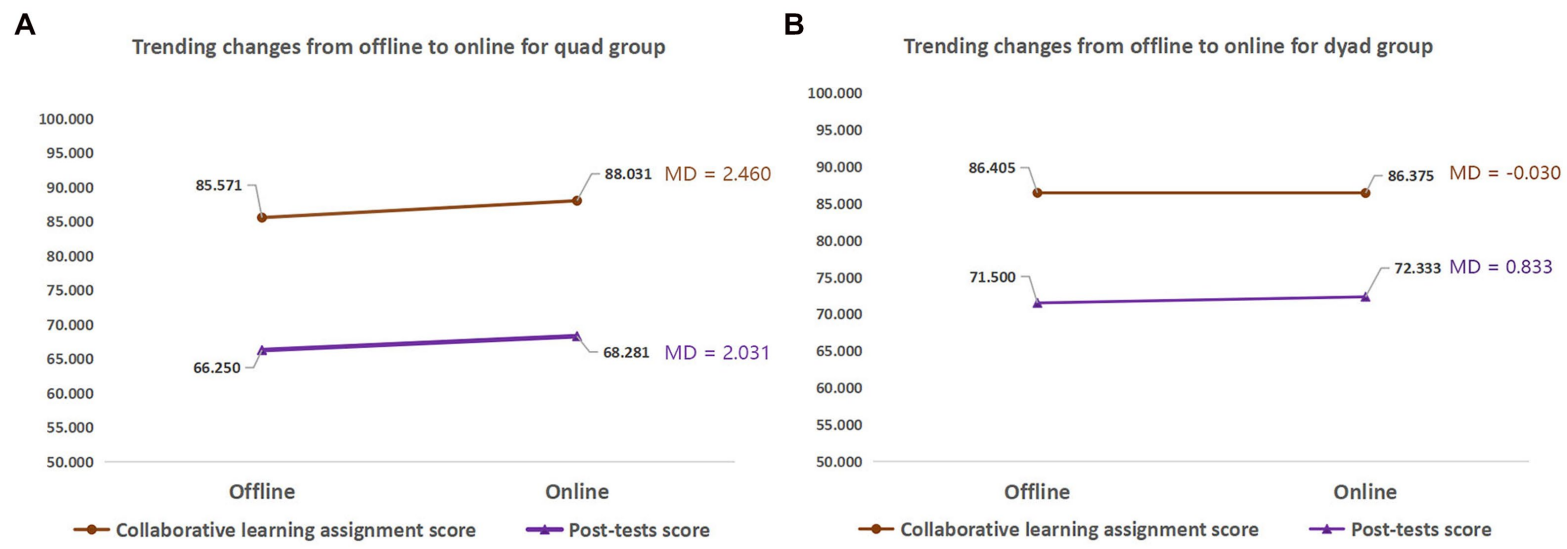
Trends of different sized groups in learning outcomes. **(A)** Quad groups; **(B)** Dyads. MD, mean difference (online score − offline score); ^*^p < 0.05; ^**^p < 0.01.

**Figure 7:**
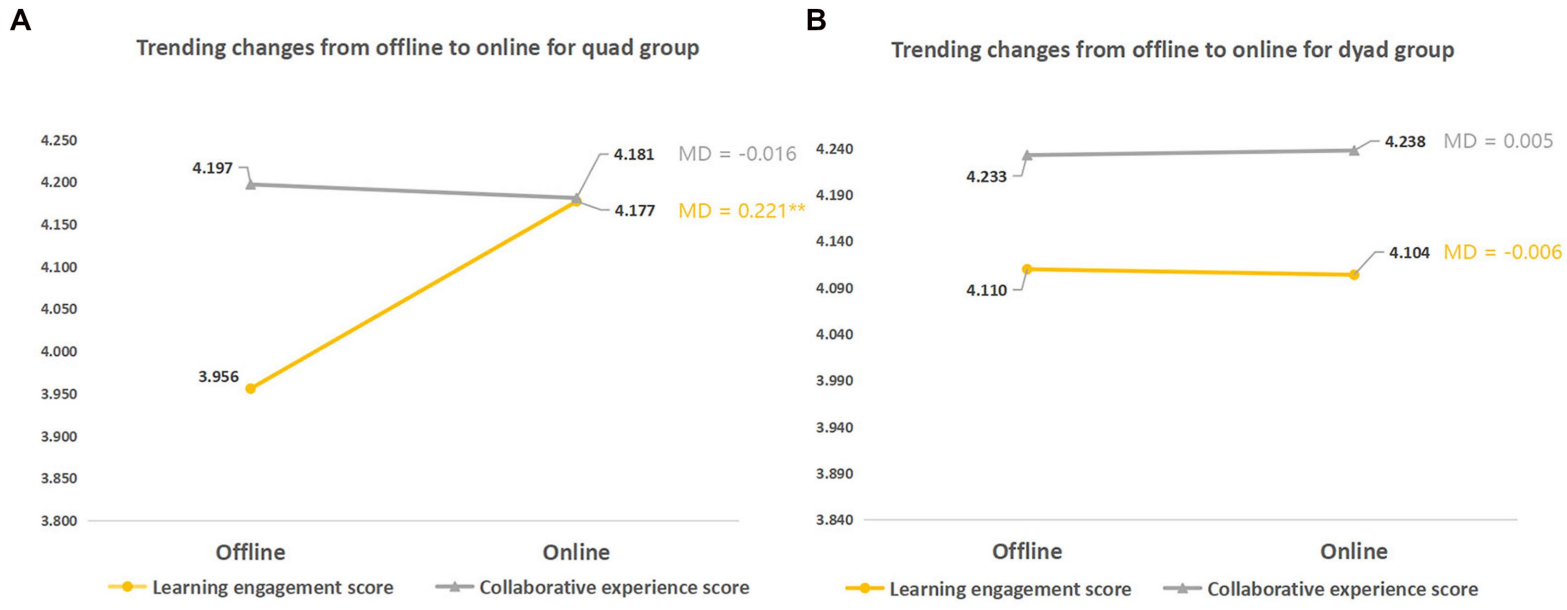
Trends of different sized groups in learning engagement and collaboration experience. **(A)** Quad groups; **(B)** Dyads. MD = mean difference (online score − offline score); ^*^p < 0.05; ^**^p < 0.01.

A change in learning context inevitably leads to differences between different-sized groups. We used a series of paired sample *t*-tests to further compare the data collected from the quad and dyad groups during the transition from the offline to online context. As shown in Figure 6, the change in learning context had little influence on the test scores and assignment scores for both the quad and dyad groups, as evidenced by the flat lines in the line chart. However, Figure 7 shows that the learning context had a moderating effect on the learning engagement of the participants from the quad groups, with online context associated with higher learning engagement. Thus far, the dyad groups witnessed no such moderating effect. In general, dyads were more stable and less likely to be influenced by the mode of learning context, while quad groups might be more suitable for online collaboration due to increased collaborative experience in such context.

**Figure 8:**
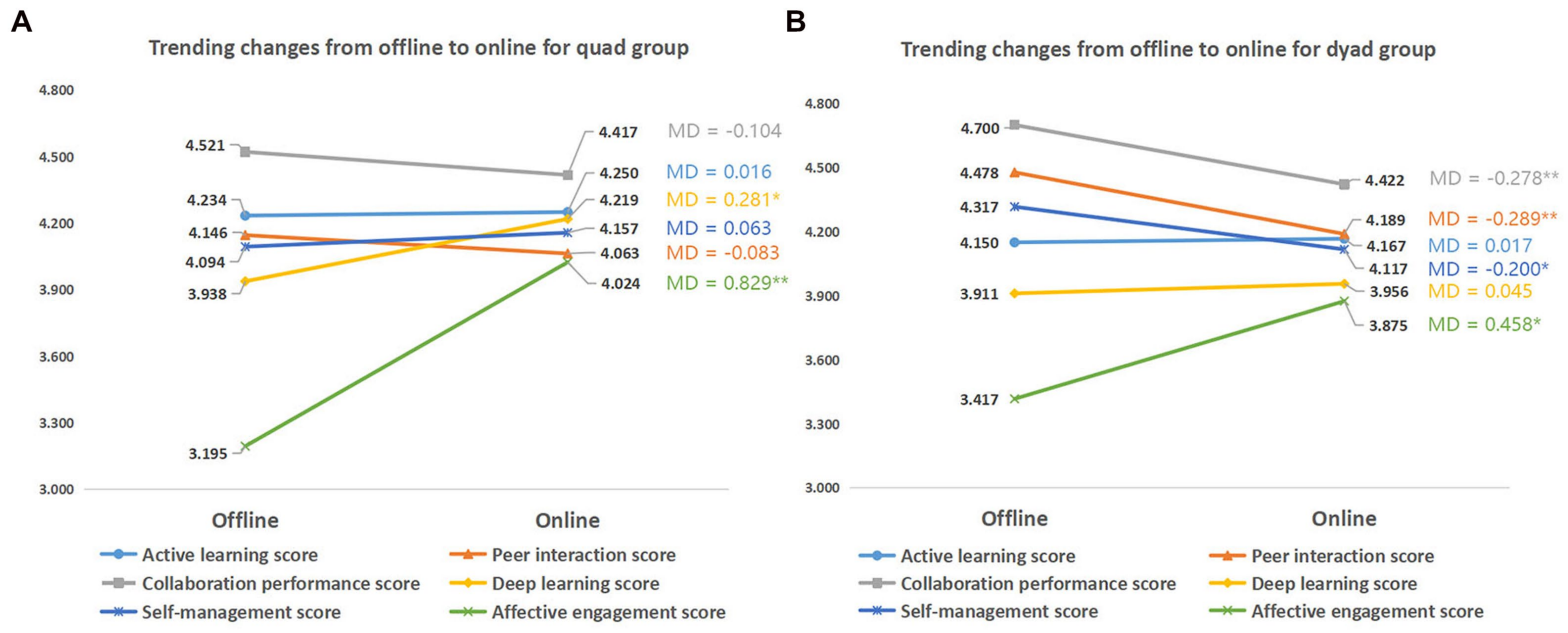
Trends in different sized groups in the learning process. **(A)** Quad groups **(B)** Dyads. MD = mean difference (online score − offline score); ^*^p < 0.05; ^**^p < 0.01.

We further examined participants’ learning engagement in terms of six subscales, namely active learning, collaboration performance, self-management, peer interaction, deep learning, and affective engagement, and compared their variance between the offline and online collaboration contexts. Figure 8 shows that the quad groups demonstrated greater affective engagement in the online collaboration context (MD = 0.829, *p* = 0.000 < 0.01) than in the offline context, and a slight improvement was found in deep learning after the online transition (MD = 0.281, *p* = 0.024 < 0.05). Similarly, the dyads reported a significant increase in affective engagement after switching to the online context (MD = 0.458, *p* = 0.010 < 0.05). However, perceived peer interaction (*p* = 0.001 < 0.01), collaboration performance (*p* = 0.004 < 0.01), and self-management (*p* = 0.043 < 0.05) witnessed a moderate decline. The results indicate that although the overall learning engagement score of dyads remained steady across the two learning contexts, variance existed in the subscales of engagement.

## 5. Discussion and conclusion

In this study, we explored the impact of changes in group size and learning context on collaborative learning, and the impact was reflected through learning outcomes, learning engagement, and collaborative experience. In general, in the same learning context, there was little difference in learning outcomes, learning engagement, and collaborative experience between quad and dyad groups. However, compared to the quad groups, dyads experienced few changes when switching contexts, which indicates that it is a more stable grouping strategy for collaborative learning.

The learning outcomes were primarily measured by collaborative tasks and post-tests. In terms of scores, except for the online case analysis report, the dyad group scored slightly higher, contrary to the findings of Shaw (2013) and Pfister and Oehl (2009). This indicates that, although the dyads were at a disadvantage in terms of numbers, they still achieved relatively better scores. One possible explanation for this is that, in the process of collaboration, each member of the dyad had more responsibility for group work. To better complete the learning task and obtain higher performance, they may have had stronger motivation, more active input, and greater participation in the process of collaboration, thus resulting in better learning performance. Contrarily, due to the increase in the number of members, the connection between individual contributions and final results in the whole process of collaboration may have been reduced, and each member’s responsibility was relatively small, which may lead to isolation and inactivity for some members (Saqr et al., 2019), as well as the phenomenon of social loafing (Aggarwal and O'Brien, 2008). The participants in the quad groups who were interviewed occasionally mentioned that a few individual members of the group did not contribute to the team’s efforts, nor did they better master the learning knowledge.

For learning engagement, we found that in offline face-to-face collaborative learning, the dyads showed more active interaction than the quad groups, which was consistent with the conclusions of Siampou et al. (2014) and van der Meijden and Veenman (2005). However, in the online collaborative learning process, there was no difference between the quad and dyad groups, which may be related to the unique benefits of the different learning context (Lee and Tsai, 2011). Offline learning provides a real environment, and peers need to communicate face to face in real time, which enable rich peer expressions, gestures, social relations, and other factors to positively affect the output and expression of personal views. In contrast, online learning generates a sense of distance; with the right level of social distance, peers are more likely to express their real ideas and regulate relationships within the group for the common goal.

To our surprise, the collaborative experience did not differ among dyad and quad groups in either the face-to-face or online contexts, contrary to the findings of Lai and Wong (2021). This shows that the change in learning context and group size had little impact on learners’ cognition and emotion from collaboration, and high satisfaction was maintained. There are three possible reasons for this. First, strong learning motivation might overcome the differences in collaborative experience caused by group size and learning context (Deci et al., 1991; Ryan and Deci, 2000). If students are interested in collaborative tasks and collaborative content, they are willing to learn whether the group size is four or two, or whether the learning context is offline or online. Second, cultural differences may also have played a part in these results (Gyasi et al., 2021); Chinese students may not have been willing to express their unhappy experiences in the questionnaire due to the influence of their own learning atmosphere. Third, the data collected by the questionnaire were in the form of self-report, which may not have been very accurate, which may make it difficult to determine the differences in the details of students’ collaborative experience.

One interesting finding of this study is that, compared with the quad groups, the dyads were relatively stable in terms of learning outcomes, learning engagement, and collaborative experience, with little changes in their scores. It seems justified to assume that the dyad group was more adaptable to the change in learning context. One possible explanation for this is that the relationship between the two group members is more equal, resulting in greater intrinsic learning motivation that was less susceptible to contextual factors (Xie and Ke, 2011). The fact that online learning context promoted learning engagement for the quad groups is consistent with the existing literature; since the online context can afford students more autonomy and flexibility while reducing social anxiety, it can promote cognitive and affective engagement for larger groups (Walsh et al., 2003; Almahasees et al., 2021).

### 5.1. Implications for teaching practice

Based on the results of the research, this paper puts forward three suggestions for implementing collaborative learning. First, for teaching large classes with a large number of students, we suggest that teachers choose groups of four for collaborative learning. Because the effects of the quad and dyad groups remained the same across different contexts, the advantages of the quad group in management efficiency could reduce teacher workload. Second, if a collaborative learning activity is primarily offline and the focus is on promoting social interaction and dialogue, then teachers are encouraged to choose dyads instead of quad groups as the former can encourage greater peer interaction due to strong real-time offline communication and equal relationships during collaboration. Finally, for the developers of online learning platforms, it is very necessary to develop a function that allows teachers to flexibly select group size and groupings according to specific teaching conditions, class size, learning tasks, and other factors.

### 5.2. Limitations and future research

Several limitations of this study need to be noted. First, the study was conducted for a short time (4 weeks), and students may have entered the next learning situation without fully grasping the characteristics of the current learning context. Therefore, in the follow-up research, the time frame for collaborative learning could be extended in the different learning contexts to improve students’ sense of the context and ensure the accuracy of the collected data. Second, the participants in this study came from the same course offered by one university and were all third-year students. Future research should improve the universality of the findings by improving the diversity of the participant groups, collaborative learning tasks, and disciplinary domains, and investigate the impact of demographics such as learners’ gender, age, academic achievements, and different learning fields on the results, and experience of collaborative learning. Third, we only collected the end results of collaborative learning in this study without in-depth analysis of the learning data generated during the learning process. Future researchers can report more information of the processes leading to the outcomes, which might provide rationale for the optimal group size. Finally, the data collected in this study were primarily quantitative data lacking in diversity, such as questionnaires and evaluation scores. However, it is difficult to triangulate and explain the statistical results without in-depth analysis of, among other aspects, learning behaviors, emotions, and attitudes in the different collaborative groups. Therefore, we suggest that qualitative surveys be used in the future to improve the credibility and interpretability of the results.

## Data availability statement

The datasets presented in this study can be found in online repositories. The names of the repository/repositories and accession number(s) can be found at: https://doi.org/10.17632/6x56rzy2wt.1.

## Ethics statement

The studies involving human participants were reviewed and approved by Institutional Review Board of Central China Normal University. The patients/participants provided their written informed consent to participate in this study.

## Author contributions

HL: conceptualization and funding acquisition. LJ and HL: methodology, writing—review and editing, and supervision. MW and HL: formal analysis and investigation. MW: writing—original draft preparation and visualization. All authors contributed to the article and approved the submitted version.

## Funding

This work was supported by the Hubei Provincial Teaching and Research Project for Higher Education, Hubei, China, grant number 2021085; Teacher Education Specialized Grant of Central China Normal University, grant number CCNUTEIII 2021-10; and the “AI + Education” Innovative Teaching Research Grant of Central China Normal University, grant number 2022XY017.

## Conflict of interest

The authors declare that the research was conducted in the absence of any commercial or financial relationships that could be construed as a potential conflict of interest.

## Publisher’s note

All claims expressed in this article are solely those of the authors and do not necessarily represent those of their affiliated organizations, or those of the publisher, the editors and the reviewers. Any product that may be evaluated in this article, or claim that may be made by its manufacturer, is not guaranteed or endorsed by the publisher.

## Abbreviations

CSCL: computer-supported collaborative learning
