# Supplementary material for: Dyads or quads? Impact of group size and learning context on collaborative learning
Source: Front Psychol. 2023 May 5;14:1168208. doi: 10.3389/fpsyg.2023.1168208 (PMC10196372; doi:10.3389/fpsyg.2023.1168208)
Supplement: Supplementary file 1 [file Table_1.DOCX]

Supplementary Material

# Appendix A. Questionnaire on learning engagement and collaborative experience

## Part I: Personal information

1. Your name.
2. Your student number.
3. Your gender.
4. Your age.
5. How many people are in your collaborative learning group?

## Part II: Learning engagement questionnaire

1. I try to understand and think about what the teacher is teaching.
2. I actively put forward my own views in collaborative and communication.
3. I share resources and information with other members.
4. When I disagree with other members, I can boldly question and raise different opinions.
5. If I have more ideas on the same issue, I supplement the previous statements of other members.
6. I complete my tasks on time in collaboration.
7. I help my group members to complete difficult tasks collaboratively.
8. I carefully listen to the opinions of others in the collaboration and communication.
9. In collaborative learning, I connect the different content I have learned.
10. In collaborative learning, I browse various learning resources with questions.
11. In collaborative learning, I use what I have learned to understand new knowledge points.
12. I make rational use of my time in collaborative learning.
13. In collaborative learning, I have clear learning objectives.
14. Collaborative learning can stimulate my enthusiasm for learning.
15. I am very interested in this course that has adopted collaborative learning.
16. I consider myself a member of the group.
17. I feel happy with the learning process of this course.

## Part III: Collaborative experience questionnaire

1. I actively participate in the collaboration.
2. I believe that all members work together for the purpose of the mission in the collaboration.
3. All members in the collaboration can equally participate in the discussion.
4. In the collaboration process, members can negotiate different views.
5. My views and questions can be answered by other members in a timely manner.
6. Members help each other.
7. I choose to resolve conflicts in discussion through consultation rather than avoidance.
8. I know the tasks to be completed through the collaboration.
9. I can keep up with the rhythm of the discussion.
10. When the discussion deviates from the theme, I consciously guide other members back to the theme.
11. Collaborative learning has promoted my understanding of the course content.
12. Collaborative learning can stimulate my interest in learning.
13. I like to learn in a collaborative way during the learning process.
14. If I have the opportunity in the future, I am willing to participate in the study of collaborative learning.

# Appendix B. Semi-structured interview protocol

Hello, classmate! Thank you very much for taking the time to talk with me after class. After two weeks of teaching practice, I would like to get more information about the learning effects of different online and offline cooperation modes from the perspective of learners.

The content of this interview will be completely recorded so that it can be analyzed in detail later. We guarantee that all of the content of the interview is completely confidential and will only be used for research.

Please talk about your true views on the following issues and try to speak freely. Thank you!

1. How many teams are in this course?

2. Can you describe your collaborative learning together? Is there anything that impresses you most?

3. What do you think of collaborative learning?

(likes, perceived effects, peer or group member evaluations, potential problems)

4. After two weeks of course learning, do you think there is a difference between the collaborative experience in the classroom and in the forum? If so, what are the particular differences?

(Communication, participation, listening, expression, division of labor)

5. How do you evaluate your group’s collaborative learning in class? Please talk about it in detail

6. On the forum, how do you evaluate your group’s collaborative learning? Please talk about it in detail.

(own/group performance, own experience, and learning promotion)

7. For future classroom learning, do you prefer online collaborative learning or offline collaborative learning? Why?

8. What are your suggestions for improving collaborative learning in class?

9. What are your suggestions for improving offline collaborative learning?
